# Supplementary material for: Powerful and Efficient Strategies for Genetic Association Testing of Symptom and Questionnaire Data in Psychiatric Genetic Studies
Source: Sci Rep. 2019 May 17;9:7523. doi: 10.1038/s41598-019-44046-0 (PMC6525248; doi:10.1038/s41598-019-44046-0)
Supplement: Supplementary file 1 — Supplementary Information [file 41598_2019_44046_MOESM1_ESM.pdf]

Powerful and Efficient Strategies for Genetic Association Testing of Symptom and Questionnaire Data in Psychiatric Genetic Studies

Aaron M. Holleman[1,2,†], K. Alaine Broadaway[3,†], Richard Duncan[3], Andrei Todor[2,3], Lynn M. Almli[4], Bekh Bradley[4,5], Kerry J. Ressler[6], Debashis Ghosh[7], Jennifer G. Mulle[2,3], Michael P. Epstein[2,3,*]

[1]Department of Epidemiology, Emory University, Atlanta, GA

[2]Center for Computational and Quantitative Genetics, Emory University, Atlanta, GA

[3]Department of Human Genetics, Emory University, Atlanta, GA

[4]Department of Psychiatry and Behavioral Sciences, Emory University, Atlanta, GA

[5]Clinical Psychologist, Mental Health Service Line, Department of Veterans Affairs Medical Center, Atlanta, GA

[6]Department of Psychiatry, McLean Hospital, Harvard Medical School, Belmont, MA

[7]Department of Biostatistics and Informatics, Colorado School of Public Health, Aurora, CO

[†] Contributed equally to this work

[*] Corresponding author

Address for Correspondence:
Michael P. Epstein, Ph.D.
Department of Human Genetics
Emory University School of Medicine
615 Michael Street, Suite 301
Atlanta, GA 30322
Phone: (404)712-8289
Email: mpepste@emory.edu

SUPPLEMENTARY INFORMATION

Supplementary Methods

Supplementary Figure S1

Supplementary Figure S2

Supplementary Figure S3

Supplementary Figure S4

Supplementary Figure S5

Supplementary Figures S6a-S6c

Supplementary Methods

Assumptions and Notation: We assume an inventory, test, or questionnaire with Q questions. The response to each question q ($q=1,\ldots,Q$) is an ordinal response ranging from 0 to F, where F is the maximum score possible. We assume a population-based sample of N subjects have responded to the questionnaire and possess common-variant data in a target gene or region. For subject j ($j=1,\ldots,N$), we define $\boldsymbol{P}_j = (P_{j,1}, P_{j,2}, \ldots, P_{j,Q})$ as subject j's responses to the Q questions. We then define a matrix of questionnaire responses for the entire sample $\boldsymbol{P} = \left(\boldsymbol{P}_1^T, \boldsymbol{P}_2^T, \ldots, \boldsymbol{P}_N^T\right)^T$, which is of dimension NxQ. Finally, for subject j, we define the traditional cumulative score typically used for genetic analysis of BDI or PSS as $\mathbf{S}_j = \sum_{q=1}^{Q} P_{j,q}$.

Similarly, we define $\boldsymbol{G}_j = (G_{j,1}, G_{j,2}, \ldots, G_{j,V})$ to be the genotypes of subject j at V SNPs, where $G_{j,v}$ is coded as the number of copies of the minor allele that the subject possesses at SNP v. The SNPs included in $\boldsymbol{G}_j$ will be referred to as the "SNP set." We then construct the matrix of genotypes for the sample as $\boldsymbol{G} = \left(\boldsymbol{G}_1^T, \boldsymbol{G}_2^T, \ldots, \boldsymbol{G}_N^T\right)^T$, which is of dimension N x V. Several approaches to constructing a SNP set have previously been described[1,2]. For demonstration purposes in this manuscript, we will define a SNP set as common variants (minor-allele frequency [MAF] > 5%) that fall within 2kb of a gene of interest.

GAMuT uses a KDC framework to perform a SNP-set test to test for independence between $\boldsymbol{P}$ (NxQ matrix of multivariate responses to a questionnaire) and $\boldsymbol{G}$ (NxV matrix of multivariate genotypes). After standardizing $\boldsymbol{P}$ and $\boldsymbol{G}$, we develop an NxN questionnaire-similarity matrix $\boldsymbol{Y}$ (based on $\boldsymbol{P}$) and a NxN genotypic-similarity matrix $\boldsymbol{X}$ (based on $\boldsymbol{G}$). The choice of how to model pairwise similarity or dissimilarity for a set of multivariate

outcomes is quite flexible. For example, for $\boldsymbol{P}$, we can model the matrix $\boldsymbol{Y}$ using a projection matrix, as suggested by Zapala and Schork[3], such that $\boldsymbol{Y} = \boldsymbol{P}(\boldsymbol{P}^T\boldsymbol{P})^{-1}\boldsymbol{P}^T$. We can also construct the model $\boldsymbol{Y}$ using user-selected kernel functions[1,4-6] such as the linear kernel, $y(\boldsymbol{P}_i,\boldsymbol{P}_j)=\sum_{l=1}^{L} P_{i,l}P_{j,l}$ or a quadratic kernel, $y(\boldsymbol{P}_i,\boldsymbol{P}_j)=(1+\sum_{l=1}^{L} P_{i,l}P_{j,l})^2$.

For genotypes $\boldsymbol{G}$, we model its corresponding matrix $\boldsymbol{X}$ using kernel functions $x(\boldsymbol{G}_i, \boldsymbol{G}_j)$ that can take the same form (e.g., linear, quadratic, Gaussian, Euclidean distance) used to construct $y(\boldsymbol{P}_i, \boldsymbol{P}_j)$, although additional kernels based on identity-by-state sharing are also possible. We may wish to further augment $x(\boldsymbol{G}_i, \boldsymbol{G}_j)$ to preferentially upweight the contributions of particular SNPs in $\boldsymbol{G}$ over others in the gene. For simulations reported here, we implement a weighting scheme based on the minor-allele frequency (MAF) of each assayed SNP that weights rarer variants over more common ones as described in Kwee et al[4]. Another possible SNP weight could be a measure of the strength of association between the SNP and some related mental-health phenotype (e.g. major depressive disorder, schizophrenia) that is available from an independent public dataset like those provided by the Psychiatric Genomics Consortium[7-9]. Using such independent external weights likely has value since it could be argued that a variant associated with a psychiatric phenotype (e.g. MDD) in one dataset is more likely to be associated with a correlated psychiatric phenotype measured by PSS or BDI in an independent dataset given existing knowledge about the shared genetic overlap among such traits[10,11]. We can construct such a SNP weight as a function of the log odds ratio of the SNP in the independent dataset. Once we determine the weight function, we then create a diagonal weight matrix $\boldsymbol{W}$= diag($w_1, w_2, \ldots, w_V$), where w_v, reflects the relative (normalized) weight for the vth variant in the gene.

Using $\boldsymbol{W}$, we can then create a weighted linear kernel function as $\boldsymbol{X}=\boldsymbol{GWG}^T$. Derivation of other weighted kernel functions is straightforward.

Once we construct the similarity matrices $\boldsymbol{Y}$ and $\boldsymbol{X}$, we derive our GAMuT approach as a test of independence between the elements of these two matrices. Briefly, we center each matrix as $\boldsymbol{Y_c}=\boldsymbol{HYH}$ and $\boldsymbol{X_c}=\boldsymbol{HXH}$. Here, $\boldsymbol{H} = (\boldsymbol{I} - 1_N 1_N^T / N)$ is a centering matrix with property $\boldsymbol{HH}=\boldsymbol{H}$, $\boldsymbol{I}$ is an identity matrix of dimension N, and 1_N is an $Nx1$ vector with each element equal to 1. Using $\boldsymbol{Y_c}$ and $\boldsymbol{X_c}$, we construct our test of independence of the two matrices as

$$T_{\text{GAMuT}} = \frac{1}{N}\text{trace}(Y_c X_c) \quad (1)$$

Under the null hypothesis of independence of the two matrices, T_{GAMuT} follows the same asymptotic distribution as

$$\frac{1}{N^2}\sum_{i,j=1}^{N}\lambda_{X,i}\lambda_{Y,j}z^2_{ij} \quad (2)$$

where $\lambda_{X,i}$ is the ith ordered eigenvalue of $\mathbf{X_c}$, $\lambda_{Y,j}$ is the jth ordered eigenvalue of $\mathbf{Y_c}$, and z^2_{ij} are independent and identically-distributed χ^2_1 variables[12]. We derive *P*-values for our GAMuT test analytically using Davies' exact method[13], which is a computationally efficient method to provide accurate *P*-values in the extreme tails of tests that follow mixtures of chi-square variables[6]. An implementation of Davies' method is available in the R library *CompQuadForm*.

<u>*Adjusting for Covariates:*</u> Genetic association tests must adjust for important covariates, such as principal components of ancestry, to avoid potential confounding of results. We can control for confounders before applying GAMuT by regressing each

symptom scale separately on covariates of interest and then using the residuals to form the phenotypic similarity matrix $\boldsymbol{Y}$. Although residualizing categorical phenotypes is not standard, studies have suggested that this procedure does not affect the validity of genetic association tests in case-control studies[14,15]. As we describe in the Results section, such residualization provides an effective correction for confounders within our simulated ordinal datasets.

Simulations: We conducted simulations to verify that GAMuT properly preserves type-I error (i.e., empirical size) and to assess power of GAMuT relative to standard association tests that treat questionnaire responses as a univariate outcome variable resulting from summing the responses into a continuous score. We perform simulations based on SNPs and LD patterns located within 2 kb up- and down-stream from two genes: *signal transducer and activator of transcription 3 (STAT3)*, a gene on chromosome 17q21.31, and *leucine rich repeat and fibronectin type III domain containing 5 (LRFN5)*, a gene on chromosome 14q21.1. We show the MAF and pairwise LD structure of SNPs in *STAT3* and *LRFN5* in Supplementary Figs. S1 and S2, respectively. To incorporate observed LD patterns from HapMap samples, we used the HAPGEN package[16] to generate simulated SNP data. HAPGEN generates simulated genotype information for all SNPs identified in HapMap within each gene; however, to better replicate real GWAS conditions, we applied the testing approaches only to those SNPs that would be typed on standard genotyping arrays. Although 27 common SNPs fall within the *STAT3* gene, only 14 of the 27 are genotyped on the Illumina HumanOmni1-Quad genotyping platform. Thus, the 14 typed SNPs form the SNP set for the kernel approach, and only the 14 typed SNPs are tested for association. Similarly, *LRFN5* contains 127 common SNPs, only 50 of which are typed on the Illumina

HumanOmni1-Quad array, resulting in a set of 50 SNPs tested for association in the *LRFN5* analyses. Under simulations where the causal SNP is not genotyped, power to detect an association relies on LD between the causal SNP and typed SNPs.

We simulate multivariate questionnaire data to mimic the BDI questionnaire results obtained from GTP participants. The BDI consists of 21 groups of statements that reflect various symptoms and attitudes associated with depression. Each group includes 4 statements, which correspond to a scale of 0 to 3 in terms of intensity. The 21 groups are sadness, pessimism, past failure, loss of pleasure, guilty feelings, punishment feelings, self-dislike, self-criticalness, suicidal thoughts or wishes, crying, agitation, loss of interest, indecisiveness, worthlessness, loss of energy, changes in sleep patterns, irritability, changes in appetite, concentration difficulty, fatigue, and loss of libido. The BDI is generally self-administered or self-reported, and is scored by summing the ratings given to each of the 21 items. Summing the responses yields a score ranging from 0-63, with scores higher than 28 being indicative of moderate to severe depression.

To simulate BDI data, we first generated 21 outcomes for each subject using a multivariate normal distribution with mean vector 0 and QxQ correlation matrix $\mathbf{\Sigma}$. We calculated $\mathbf{\Sigma}$ based on observed Spearman rank correlation calculations from the GTP BDI questionnaire responses shown in Supplementary Fig. S3. The observed correlations between questions ranged from 0.22 to 0.57. Next, we generated ordinal responses from the normally distributed variables to match the ordinal responses observed in GTP data. Frequency of scores by each of the 21 BDI questions is shown in Supplementary Fig. S4. We found the percent of GTP participants who answered 0, 1, 2, and 3 for each question. We then matched the percentages of each BDI question for each of the 21 normally distributed

variables. For example, in BDI Question 1 ("Sadness"), 56% of participants answered 0 ("I do not feel sad"), 34% answered 1 ("I feel sad much of the time"), 6% answered 2 ("I feel sad all of the time"), and 4% answered 3 ("I am so sad or unhappy that I can't stand it."). To simulate ordinal responses to Question 1, the lowest 56% of the continuous outcomes were assigned a score of 0, values falling in the 57-90 percentile were assigned a score of 1, 91-96 percentiles were assigned a score of 2, and values in the 97th percentile and above were assigned a score of 3. We set sample size *N* of either 1,000 or 2,500 subjects. We applied GAMuT to 10,000 null simulated datasets to estimate empirical size.

To investigate the performance of GAMuT under confounding and to assess whether the approach can successfully adjust for relevant covariates in this setting, we also simulated questions under a confounding model where question responses were independent of genotype, but both questions and genotype are associated with a continuous covariate Z. We simulated questions correlated with the covariate Z under the model $\mathbf{P} \sim MVN(0.2\mathbf{Z}, \Sigma)$, where **Z** denotes the *N* x 1 sample vector of covariates. We arbitrarily selected SNP 9 (rs9909659) in the *STAT3* gene as causally associated with the confounder. We simulated correlation between SNP 9 and the covariate by generating the effect size of confounder on SNP as $\beta_Z=0.2$. Testing empirical size under this model allows us to verify that our approach to control for confounders is valid.

For power models, we simulated data sets in which each of the SNPs within *STAT3* and *LRFN5* were modeled as being causal (i.e., each of the 27 SNPs within *STAT3* was modeled as causal, and each of the 127 SNPs within *LRFN5* was set as causal). We model effect size of the causal SNP on each question, β_q, as $\beta_q = N(0.1, 0.03)$. This formulation sets mean effect sizes with modest effect on the overall cumulative score; for a causal SNP with

MAF=0.3, this formulation corresponds to an R^2=0.009 when the SNP is associated with all questions in the questionnaire. Allowing β_q to vary around a normal distribution allows the variant to have a slightly different effect size for each question. We also vary the number of questions that are associated with the causal SNP, such that not all of the questions will be dependent on the gene of interest. We consider situations where 18/21, 12/21, and 6/21 questions are actually associated with the causal SNP. We control residual correlation among questions through consideration of trait-specific heritability (i.e., the relative variance of $\mathbf{P}_q$ explained by the causal SNP). We define trait-specific heritability for question q as $\mathrm{h}_q = \beta^2_{SNP,q} * 2\mathrm{MAF}_{SNP}(1\text{-}\mathrm{MAF}_{SNP})$, where MAF_{SNP} is the MAF of the causal SNP. The correlation between questions q and q' is defined as $E_{q,q'} = \sqrt{1-h_q}\sqrt{1-h_{q'}} * \mathbf{\Sigma}_{q,q'}$ where $\mathbf{\Sigma}$ is the LxL residual correlation matrix shown in Supplementary Fig. S3. This allows the residual correlation structure among phenotypes to stay at the defined values.

Using the simulated data, we evaluated GAMuT using either projection matrices or linear kernels to model phenotypic similarity and using weighted linear kernels to model genotypic similarity (with weights based on sample MAF). We compare GAMuT to two standard approaches that use the univariate cumulative questionnaire score for inference. First, we consider a standard linear regression model that follows the form

$$\boldsymbol{S} = \mathbf{Z}\gamma + \beta_{SNP}\mathbf{G}_m + e \qquad (3)$$

where $\boldsymbol{S}$ is the Nx1 vector of cumulative scores, $\boldsymbol{Z}$ is an N x c vector of c covariates (including an intercept) with regression parameter vector γ, $\boldsymbol{G}_\mathbf{m}$ denotes an Nx1 vector of SNP genotypes at SNP m with regression parameter β_{SNP}, and the residual error e follows a

MVN distribution, $e \sim MVN(0, \sigma^2 \boldsymbol{I})$, where $\boldsymbol{I}$ denotes the *NxN* identity matrix. We then implement a likelihood ratio test to assess the null hypothesis of $\mathrm{H}_0 : \beta_{\mathrm{SNP}} = 0$ for each SNP *m*. To adjust for multiple testing of *M* correlated SNPs, we apply P_{ACT}[17] to the smallest observed *P-value*.

As power differences between GAMuT and the linear regression approach mentioned in the previous paragraph could be due either to joint modeling of multivariate questionnaire data over cumulative score or joint modeling of multiple genetic variants over a single variant, we further teased these factors apart by considering an additional gene-based test of the cumulative sum ***S*** using a kernel-machine regression (KMR[4]) test (which is analogous to the popular SKAT[6] test but for common variants). KMR, like GAMuT, models genotypic similarity using a weighted linear kernel with weights based on sample MAF. Since GAMuT and univariate KMR both consider analyses on the gene-based level, comparison of the two approaches should help highlight the benefit of considering a multivariate questionnaire phenotype over a traditional cumulative-based score for analysis.

Supplementary Figure S1: Pairwise LD (R^2) heatmap and MAF for all SNPs reported in HapMap ±2kb of the *STAT3* gene. MAF is plotted below, with genotyped SNPs denoted by the 'x' on the bottom of the MAF plot.

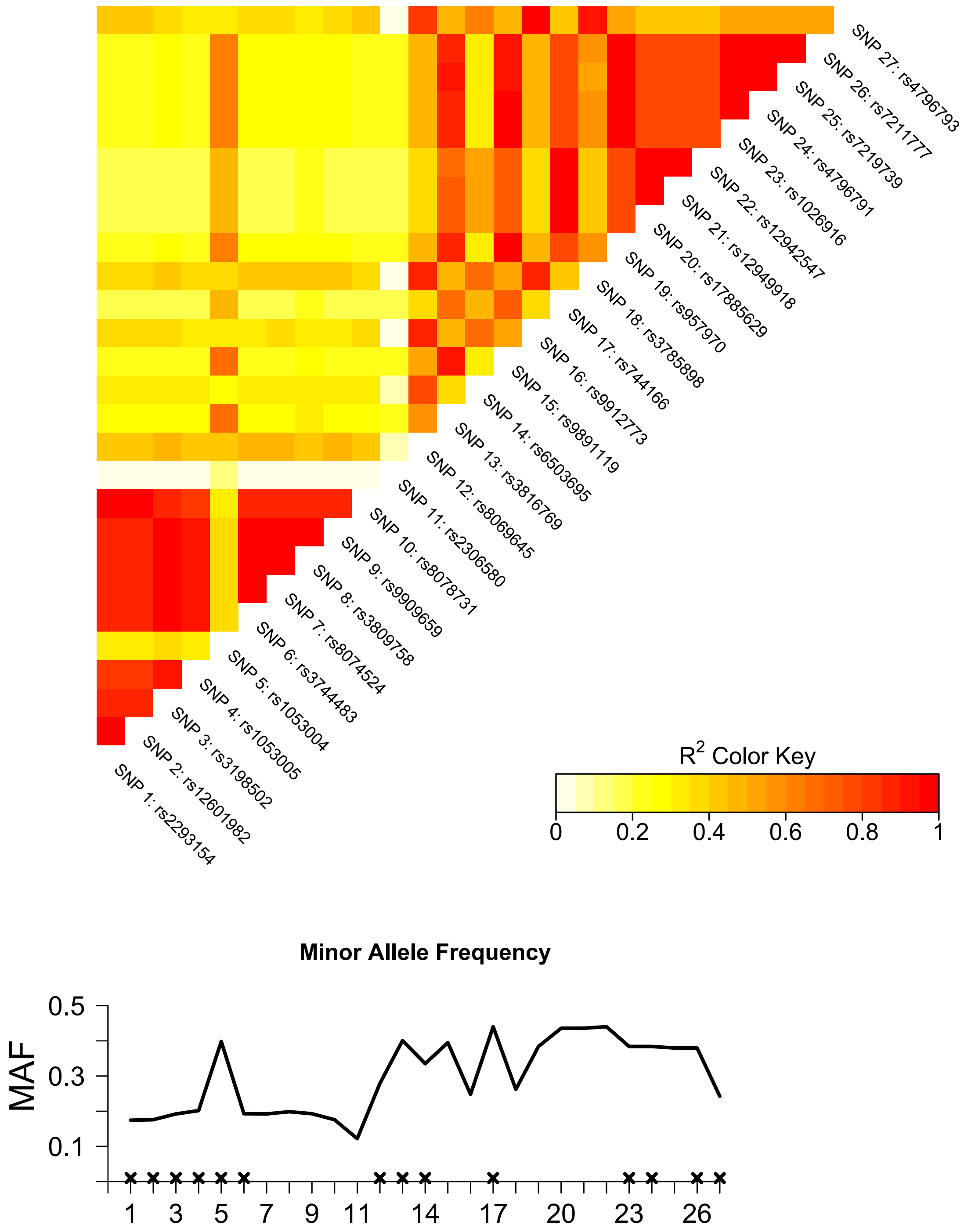

Supplementary Figure S2: Pairwise LD (R^2) heatmap and MAF for all SNPs reported in HapMap ±2kb of the *LRFN5* gene. MAF is plotted below, with genotyped SNPs denoted by the 'x' on the bottom of the MAF plot.

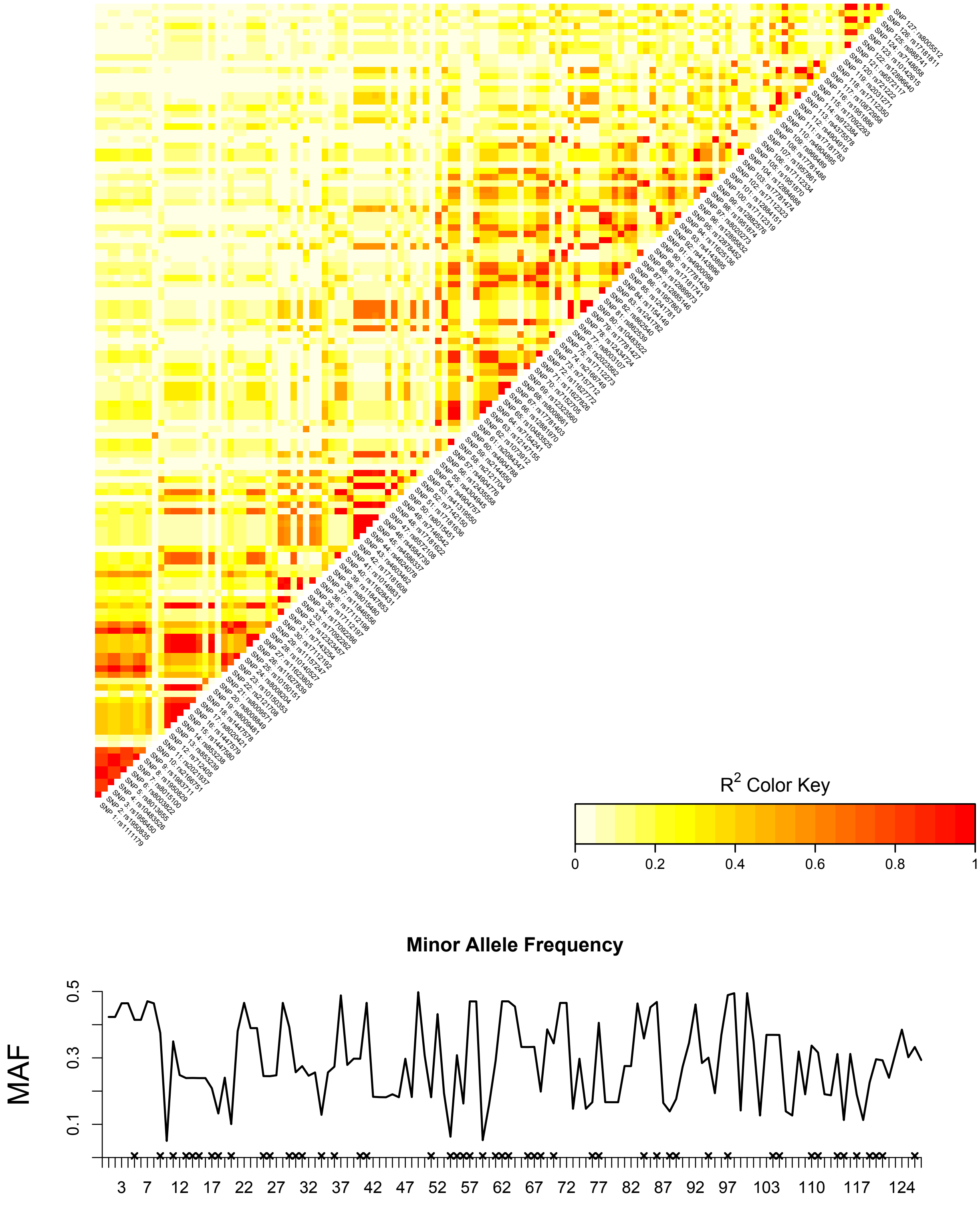

Supplementary Figure S3: Correlation among the 21 BDI question responses in the GTP dataset, after adjusting for covariates. Dark green indicates correlation of 0.6 while white indicates correlation of 0.2. Correlation among all questions was positive. All correlations are significant (Pearson's product-moment correlation *P*-value < $1x10^{-15}$).

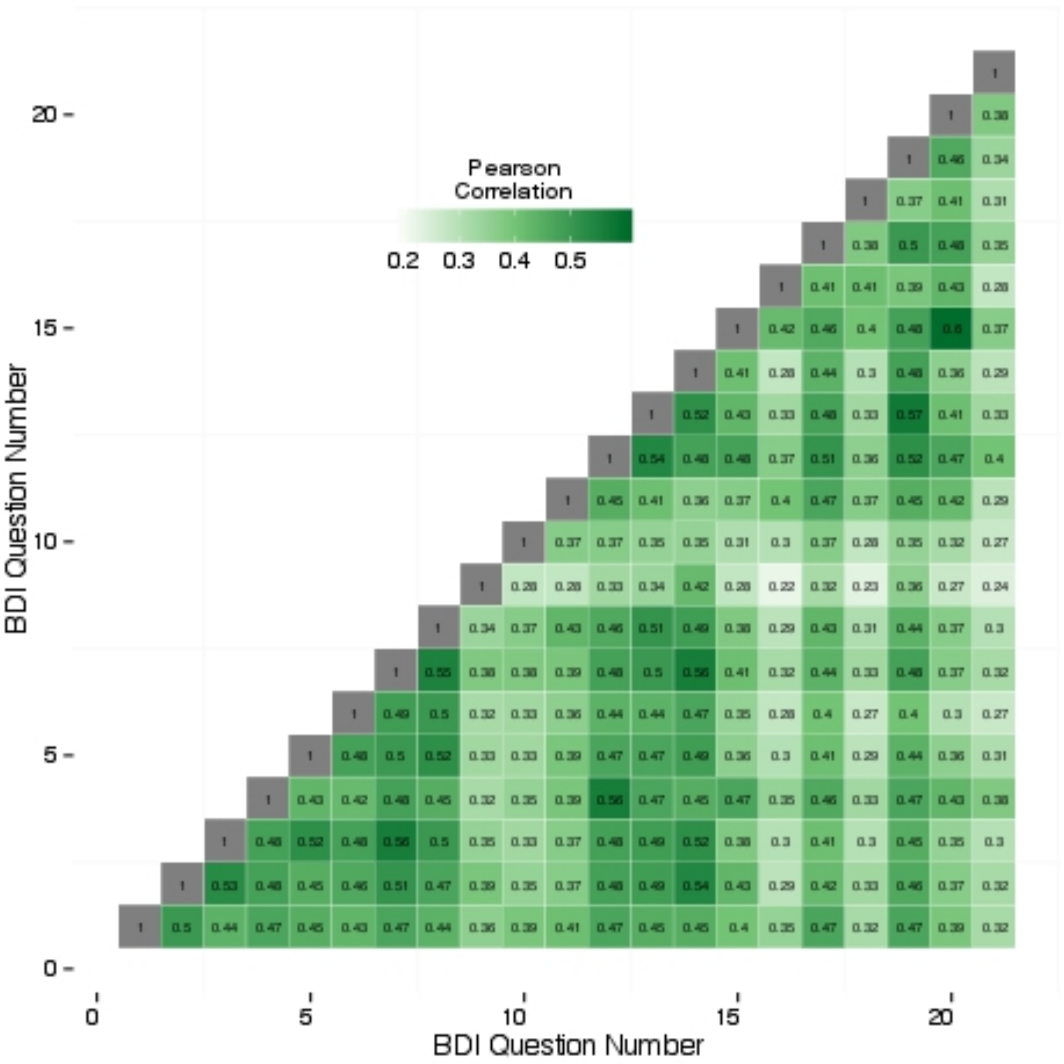

Supplementary Figure S4: Frequency of scores for each of the 21 BDI questions in the GTP dataset. The answers to each question are scored from 0 (no symptoms) to 3 (severe symptoms).

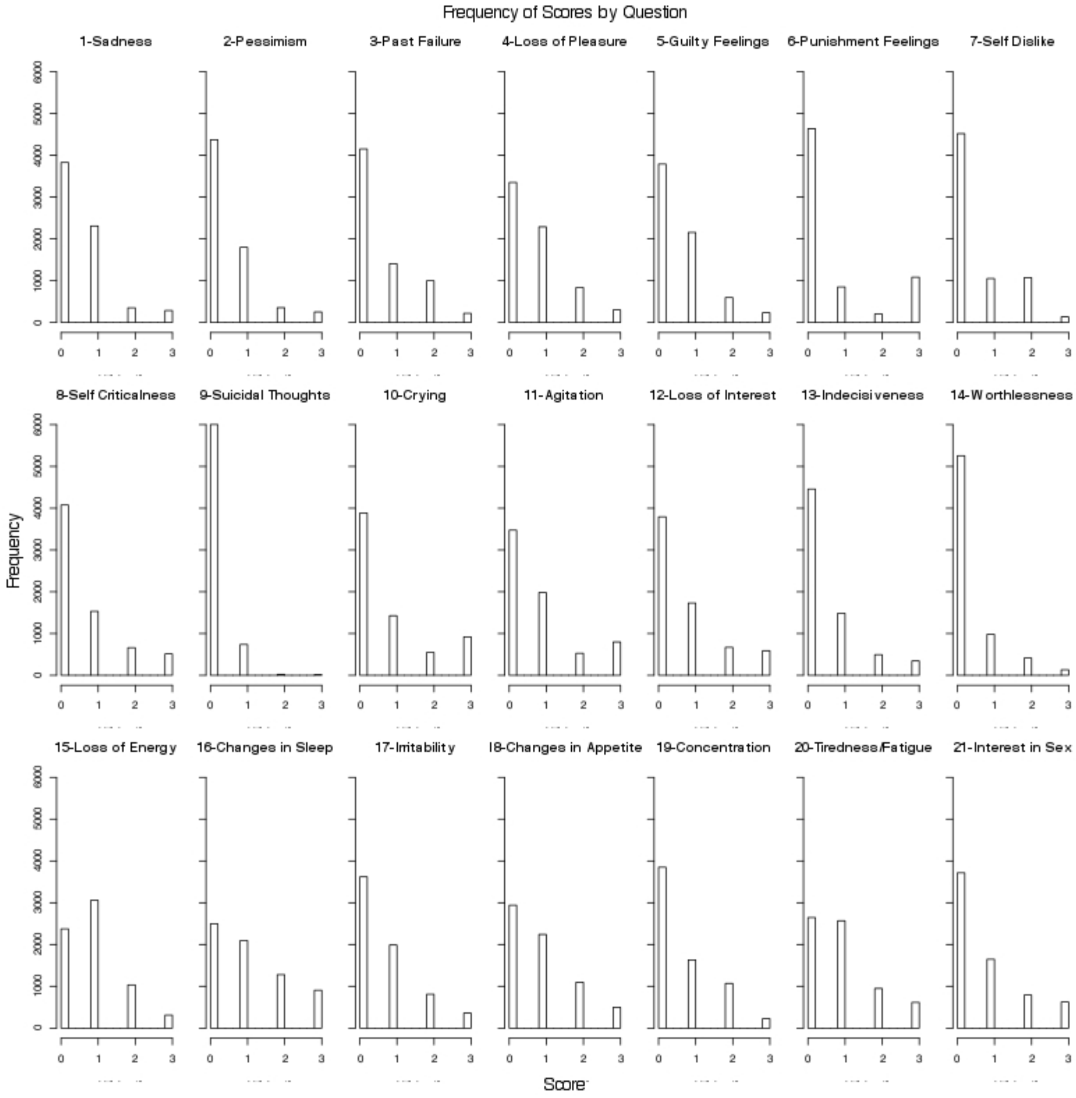

Supplementary Figure S5: The QQ plots of 10,000 simulated null datasets assuming a sample size of 1,000 with a confounding variable. Questionnaire responses are independent of genotypes (for SNPs in *STAT3*), but both responses and genotypes are associated with a continuous covariate. Left shows QQ plots without adjustment for confounding, while right shows QQ plots after adjustment for confounding by residualization.

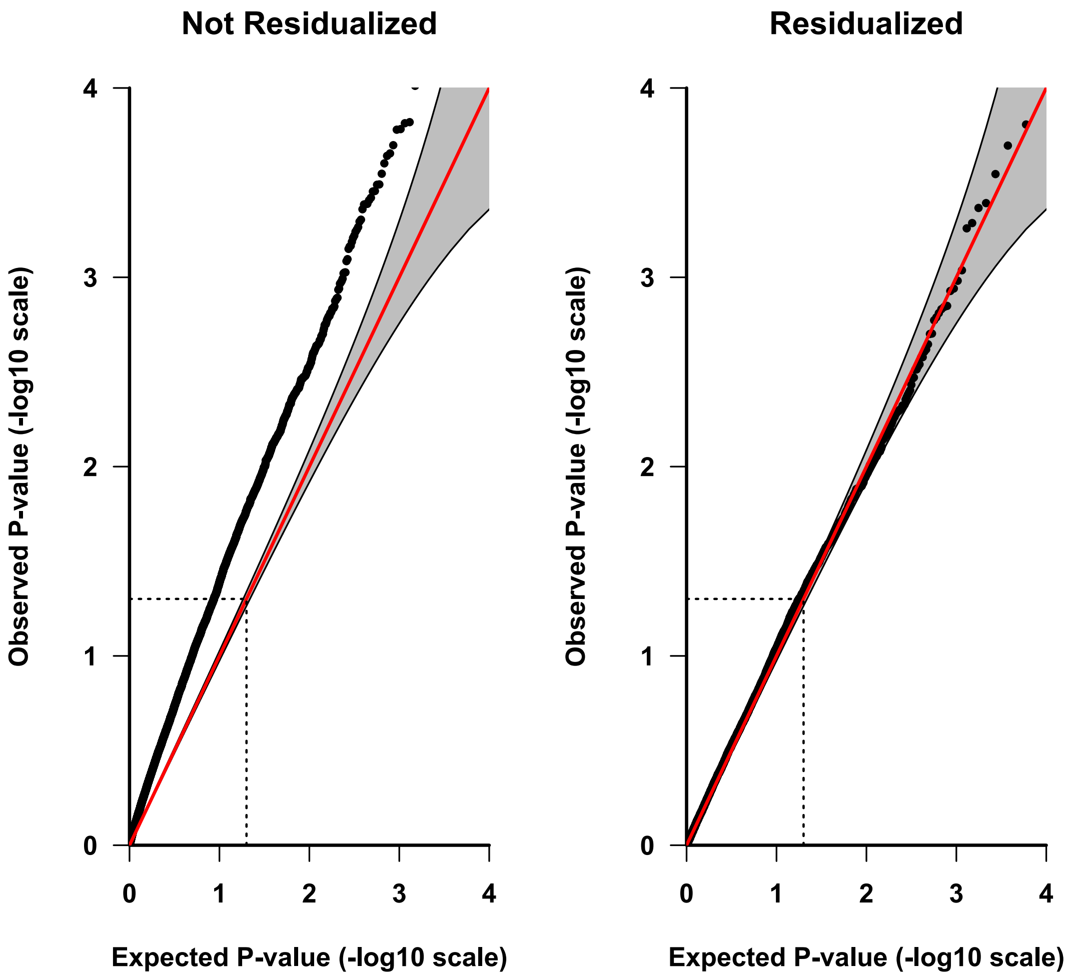

Supplementary Figures S6a-S6c: Application of GAMuT, univariate KMR, and standard linear regression to BDI (21 items). Supplementary Figure S6a includes plots for the GAMuT analyses (which used a linear kernel for modeling phenotypic similarity), showing the results for each genotype weighting method. In the Manhattan plots, the red line represents the study-wise significance threshold (based on a Bonferroni correction for the number of genes tested), and the blue line represents the suggestive significance threshold. Supplementary Figures S6b and S6c show results from the corresponding univariate KMR (gene-level testing) and linear regression (SNP-level testing) analyses.

Supplementary Figure S6a: BDI (21 items), GAMuT

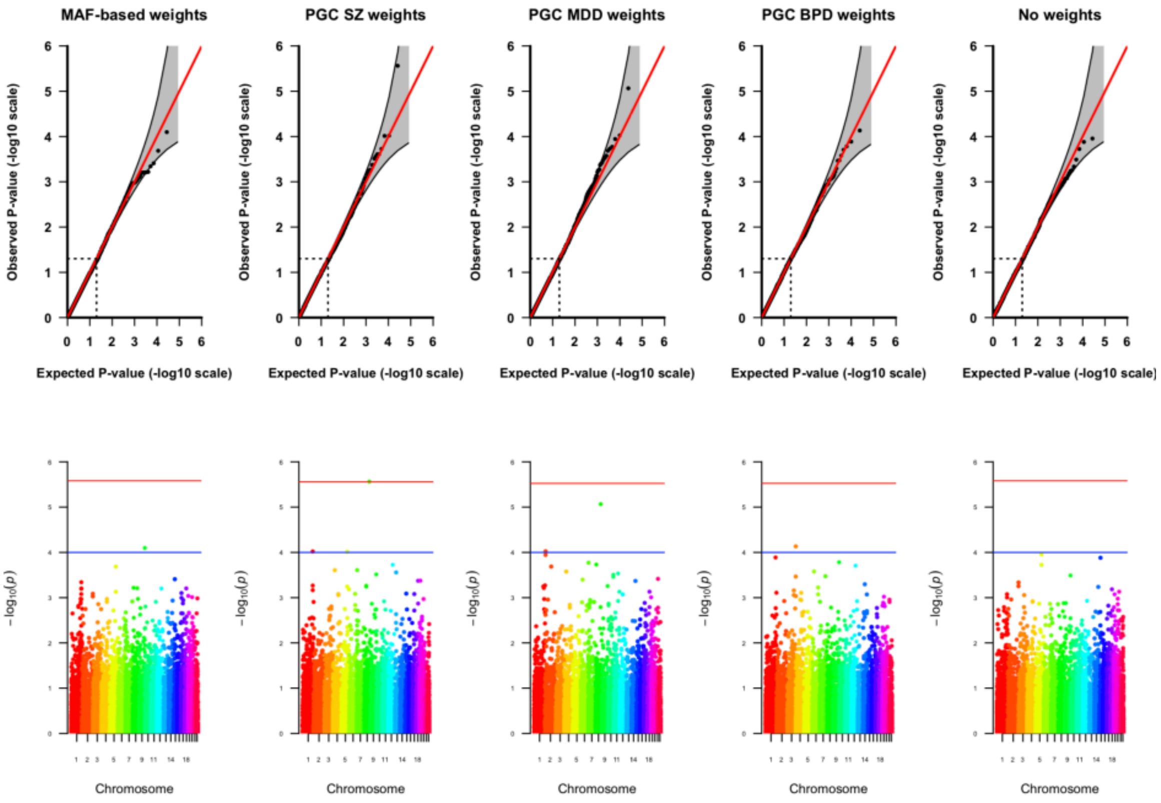

Supplementary Figure S6b: BDI (cumulative score), univariate KMR

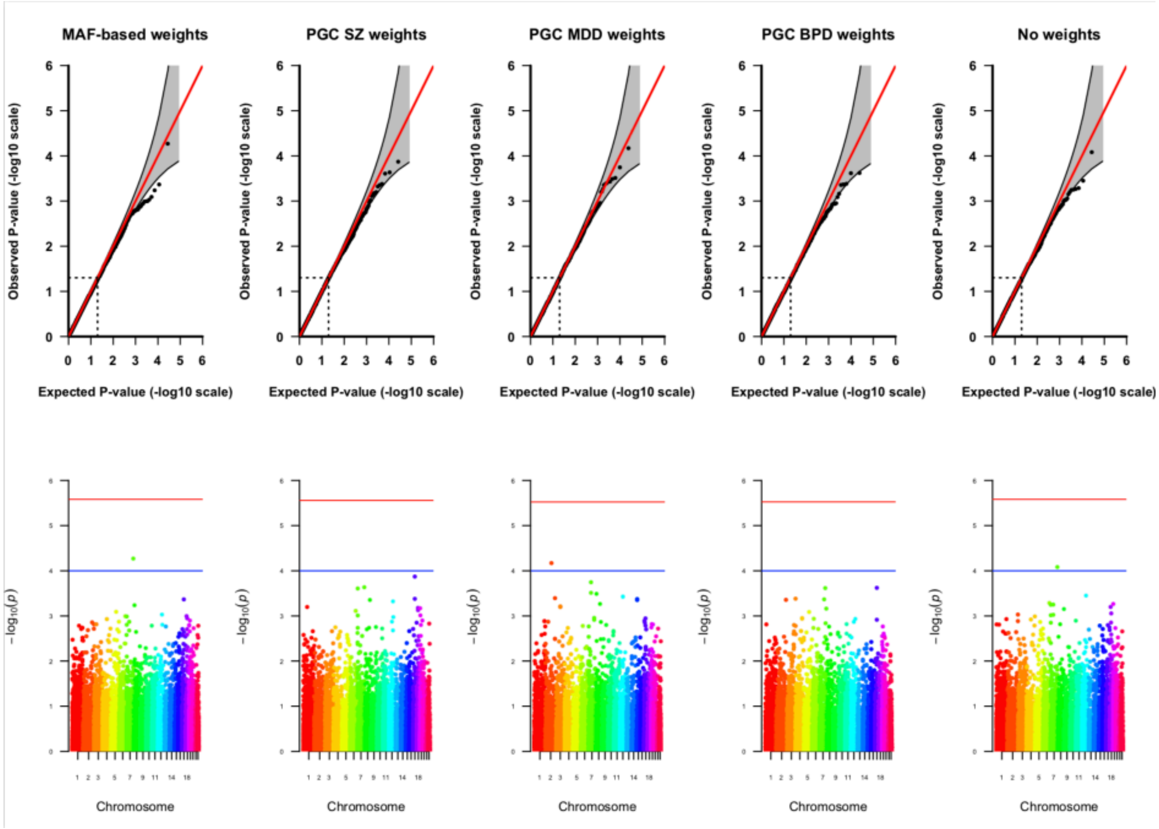

Supplementary Figure S6c: BDI (cumulative score), standard linear regression

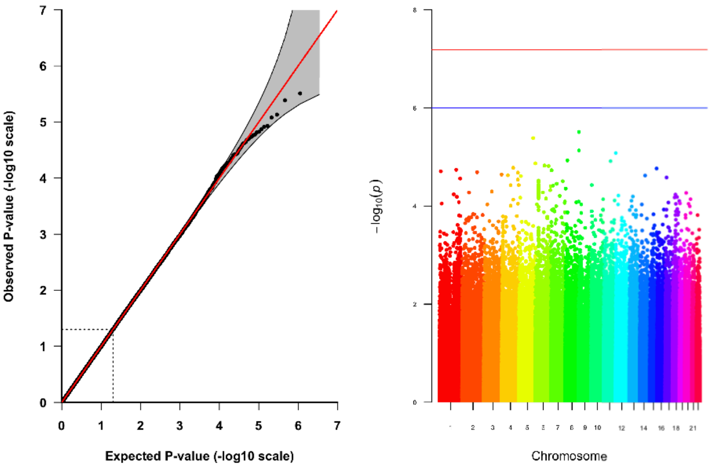

REFERENCES

1 Wu, M. C. *et al.* Powerful SNP-set analysis for case-control genome-wide association studies. *Am J Hum Genet* **86**, 929-942, doi:10.1016/j.ajhg.2010.05.002 (2010).

2 Schifano, E. D. *et al.* SNP set association analysis for familial data. *Genet Epidemiol*, doi:10.1002/gepi.21676 (2012).

3 Zapala, M. A. & Schork, N. J. Statistical properties of multivariate distance matrix regression for high-dimensional data analysis. *Front Genet* **3**, 190, doi:10.3389/fgene.2012.00190 (2012).

4 Kwee, L. C., Liu, D., Lin, X., Ghosh, D. & Epstein, M. P. A powerful and flexible multilocus association test for quantitative traits. *Am J Hum Genet* **82**, 386-397, doi:10.1016/j.ajhg.2007.10.010 (2008).

5 Schaid, D. J. Genomic similarity and kernel methods II: Methods for genomic information. *Hum Hered* **70**, 132-140, doi:10.1159/000312643 (2010).

6 Wu, M. C. *et al.* Rare-variant association testing for sequencing data with the sequence kernel association test. *Am J Hum Genet* **89**, 82-93, doi:10.1016/j.ajhg.2011.05.029 (2011).

7 Major Depressive Disorder Working Group of the Psychiatric GWAS Consortium. A mega-analysis of genome-wide association studies for major depressive disorder. *Mol Psychiatry* **18**, 497-511, doi:10.1038/mp.2012.21 (2013).

8 Psychiatric GWAS Consortium Bipolar Disorder Working Group. Large-scale genome-wide association analysis of bipolar disorder identifies a new susceptibility locus near ODZ4. *Nat Genet* **43**, 977-983, doi:10.1038/ng.943 (2011).

9 Schizophrenia Working Group of the Psychiatric Genomics Consortium. Biological insights from 108 schizophrenia-associated genetic loci. *Nature* **511**, 421-427, doi:10.1038/nature13595 (2014).

10 Duncan, L. E. *et al.* Largest GWAS of PTSD (N=20 070) yields genetic overlap with schizophrenia and sex differences in heritability. *Mol Psychiatry*, doi:10.1038/mp.2017.77 (2017).

11 Lee, S. H. *et al.* Genetic relationship between five psychiatric disorders estimated from genome-wide SNPs. *Nat Genet* **45**, 984-994, doi:10.1038/ng.2711 (2013).

12 Zhang, K., Peters, J., Janzing, D. & Schölkopf, B. Kernel-based conditional independence test and application in causal discovery. *arXiv:1202.3775v1 [cs.LG]* (2012).

13 Davies, R. B. Algorithm AS 155: the distribution of a linear combination of 2 random variables. *Journal of the Royal Statistical Society Series C Applied Statistics* **29**, 323-333 (1980).

14 Price, A. L. *et al.* Principal components analysis corrects for stratification in genome-wide association studies. *Nat Genet* **38**, 904-909, doi:10.1038/ng1847 (2006).

15 Kang, H. M. *et al.* Variance component model to account for sample structure in genome-wide association studies. *Nat Genet* **42**, 348-354, doi:10.1038/ng.548 (2010).
16 Spencer, C. C., Su, Z., Donnelly, P. & Marchini, J. Designing genome-wide association studies: sample size, power, imputation, and the choice of genotyping chip. *PLoS Genet* **5**, e1000477, doi:10.1371/journal.pgen.1000477 (2009).
17 Conneely, K. N. & Boehnke, M. So many correlated tests, so little time! Rapid adjustment of P values for multiple correlated tests. *Am J Hum Genet* **81**, 1158-1168, doi:10.1086/522036 (2007).
